# NETosis in Psoriatic Arthritis: Serum MPO–DNA Complex Level Correlates With Its Disease Activity

**DOI:** 10.3389/fimmu.2022.911347

**Published:** 2022-06-14

**Authors:** Borui Li, Guangtao Li, Xinlei Yang, Zhibo Song, Yu Wang, Zhuoli Zhang

**Affiliations:** Rheumatology and Clinical Immunology Department, Peking University First Hospital, Beijing, China

**Keywords:** psoriatic arthritis, NEtosis, MPO–DNA complex, disease activity, therapeutic response

## Abstract

**Background:**

Neutrophil extracellular trap formation (NETosis) has been rarely reported in psoriatic arthritis (PsA). We aimed to explore the involvement of NETosis in the inflammation of PsA.

**Methods:**

Serum myeloperoxidase–DNA (MPO-DNA) complex was detected by a modified enzyme-linked immunosorbent assay and compared among 74 patients with PsA, 58 patients with psoriasis (PsO), and 20 healthy controls. The association of MPO–DNA level with disease activity index at baseline and follow-up was analyzed in patients with PsA. Receiver operating characteristic curve was used to evaluate the predictive value of MPO–DNA for treatment response.

**Results:**

MPO–DNA complex level in serum was significantly increased in patients with PsA/PsO compared to healthy controls (*p* < 0.001). The level of MPO–DNA was positively associated with DAPSA score and its components (including TJC, SJC, PGA, VAS-pain and CRP, r = 0.25–0.409, all *p*-values < 0.05). Serum MPO–DNA level was downregualted at 12 weeks after treatment compared to baseline (*p* = 0.022). The decrease of MPO–DNA level was more dramatic in patients with PsA who achieved both ACR50 and PASI50 response than those achieving neither of them at 12 weeks (*p* = 0.023). ROC analysis revealed that the serum MPO–DNA level predicted both ACR50 and PASI50 achievement at week 12 (*p* = 0.04; 95% CIs, 0.56–0.94). Moreover, the baseline MPO–DNA level (*p* = 0.009; 95% CIs, 0.748–1) and change of MPO–DNA at week 12 from baseline (*p* = 0.004; 95% CIs, 0.802–1) were associated with the achievement of both ACR70 and PASI75 response at week 24.

**Conclusions:**

NETosis plays an important role in psoriatic diseases. The level of MPO–DNA complex in serum reflects disease activity. Serum MPO–DNA complex may be a useful biomarker to predict the therapeutic response in PsA.

## Background

Psoriasis (PsO) is a common chronic inflammatory skin disease that affects approximately 0.14% of the population in China (1). About one-third of patients with PsO develop psoriatic arthritis (PsA) presenting as dactylitis, enthesitis, synovitis, and so on (2). A large number of immune cells and molecules contribute to the inflammation process of PsA. Previous studies have revealed that activated T cells and monocytes may play important roles in the skin and articular lesions of PsA (3, 4). Polymorphonuclear cells also participate in the pathogenesis of PsO/PsA; however, the exact mechanism has not been fully understood yet (5).

Neutrophils play important roles in the pathogenesis of many autoimmune diseases. Previous reports have shown their involvement in PsO/PsA and implied the neutrophil extracellular trap formation (NETosis) may take part in the pathogenesis of PsO/PsA (6, 7). NETosis, reflecting a response of innate immune system to pathogens, is a key process in many autoimmune diseases such as systemic lupus erythematosus, anti-neutrophil cytoplasmatic antibody (ANCA)–associated vasculitis, and PsO diseases (8–10). Frasca et al. found that NETosis could be a source of autoantigen contributing to PsA pathogenesis (11). However, few studies ever focused on the clinical values of NETosis remnants in PsA so far. Myeloperoxidase–DNA (MPO–DNA) complex, one of the neutrophil extracellular trap components, has been detected in serum to reflct the level of NETosis in many inflammatory diseases (12, 13). In this study, we evaluated the serum level of MPO–DNA complex and explored its association with disease activity and therapeutic response in patients with PsA.

## Methods

### Study Design and Patients

This was a longitudinal study. Patients with PsA/PsO were recruited from the Rheumatology and Clinical Immunology Department of the Peking University First Hospital from June 2018 to October 2020. Patients with PsA fulfilled the Classification Criteria for PsA (CASPAR) with age above 18 years (14). Patients with other autoimmune diseases or non-psoriatic inflammatory arthritis were excluded. The clinical and laboratory profiles, including tender joint count (TJC), swollen joint count (SJC), patient’s global assessment (PGA, 0–100), visual analog scale-pain (VAS-pain) (0–100), erythrocyte sedimentation rate (ESR), and C-reactive protein (CRP) were recorded in patients with PsA. PsO area and severity index (PASI) was calculated for patients with PsA/PsO. Medications used were also collected.

### Evaluation of Clinical Disease Activity and Treatment Response in Patients With PsA

Disease activity in psoriatic arthritis (DAPSA) score was used to describe the activity of PsA (15, 16). DAPSA score was calculated by summing up the following variables: SJC, TJC, PGA, VAS-pain, and CRP. Clinical DAPSA (cDAPSA) was defined as DAPSA without CRP. The cutoff values of DAPSA score were proposed as ≤ 4 for remission, >4 and ≤14 for low disease activity (LDA), >14 and ≤ 28 for moderate disease activity (MDA), and >28 for high disease activity (HDA). The achievement of minimal disease activity or not was also evaluated at each time point (15, 16). The minimal disease activity was defined as meeting five of the following criteria: TJC ≤ 1, SJC ≤ 1, PASI ≤ 1 or body surface area ≤ 3, VAS-pain ≤ 15, PGA ≤ 20, health assessment questionnaire ≤ 0.5, and tender entheseal points ≤ 1.

For those patients with PsA who were longitudinally followed up after treatment, the therapeutic responses were recorded as American College of Rheumatology (ACR) response criteria and percentage of reductions of PASI score (17, 18). ACR20/50/70 was defined by the following three conditions: at least 20/50/70% improvement of TJC and SJC and at least 20/50/70% improvement in three of five additional domains (PGA, EGA, VAS-pain, HAQ, and ESR/CRP). PASI 50/75/70/100 were defined as 50/75/90/100% improvement of PASI score.

### ELISA Analysis of MPO–DNA Complex

Serum samples obtained from patients with PsA/PsO and healthy controls were stored at −80°C until analysis. MPO–DNA complexes were identified using a capture enzyme-linked immunosorbent assay (ELISA) (19). Anti-MPO monoclonal antibody (Abcam) 5 µg/ml, used as the capturing antibody, was coated to a 96-well plate overnight at 4°C. After blocking with %1 bovine serum albumin, serum samples were added to distinct wells (100 µl per well) for 2-h incubation at 37°C. After washing, a commercial horseradish peroxidase–labeled anti-human DNA monoclonal antibody (component no. 2 of the Cell Death Detection Kit, Roche) was added to each well, followed by a 2-h incubation at room temperature. Then, the plate was rewashed and added with the peroxidase substrate followed by a 2N sulfuric acid stop solution. The optical density (OD) of each well was subsequently measured at a wavelength of 405 nm (OD_405_), with 490 nm used as a reference. The results were expressed as mean OD_405/490_ values.

### Statistical Analysis

Data were presented as mean (SD), median interquartile range (IQR), or proportion (%) as appropriate. Comparisons of continuous variables with normal distribution such as age were performed using student’s T-test. Other continuous variables not fulfilling normal distribution were evaluated by the Mann–Whitney U-test. Categorical variables were reported as frequencies and analyzed by the Chi-square test. Pearson’s correlation was used to analyze the linear association. The predictive power of the model was accessed by measuring the area under the receiver operating characteristic (ROC) curve. The cutoff value was determined by the ROC with the highest Youden index. Results of binary logistic regression analysis were exhibited by odds ratio (OR) and 95% confidence intervals (CIs). Statistical significance was analyzed by SPSS version 26.0 software and Graphpad Prism V 8.00.

## Results

### Baseline Demographics and Cinical Features of Psoriasis and Psoriatic Arthritis at Baseline

A total of 74 patients with PsA, 58 patients with PsO, and 20 healthy controls were recruited in this study. Their age [47.0 (13.0) vs. 44.9 (12.4) vs. 49.3 (7.5)] and gender proportion (56.7% vs. 48.2% vs. 50%) were similar among all three groups (*p* > 0.05 for all). The duration of skin lesions [median (IQR) 13.0 (8.1, 20.5) vs. 13 (7.0, 20.0) years] at the first visit, ESR [median (IQR) 11.0 (4.0, 25.0) vs. 9.5 (5.0, 15.0) mm/h], CRP [median (IQR) 3.6 (2.1, 13.9) vs. 3.8 (1.9, 5.9) mg/L], and PASI [median (IQR) 2.2 (0.3, 5.0) vs. 1.8 (0.7, 4.0) years] were comparable between patients with PsA and PsO (**Table 1**).

**Table 1:** Baseline characteristics of patients with PsA and PsO.

Item	PsA (n = 74)	PsO (n = 58)
Male, n (%)	42(56.7%)	28 (48.2%)
Age, mean (SD), years	47.0 (13.0)	44.9 (12.4)
PsO duration, years	13.0 (8.1, 20.5)	13.0 (7.0,20.0)
PsA duration, years	4.5 (1.4, 9.7)	–
ESR, mm/h	11.0 (4.0, 25.0)	9.5 (5.0, 15.0)
CRP, mg/L	3.6 (2.1, 13.9)	3.8 (1.9, 5.9)
Psoriasis, n (%)	70 (94.5%)	58 (100%)
PASI, median (IQR)	2.2 (0.3, 5.0)	1.8 (0.7, 4.0)
PGA (0–100 mm)	30 (20, 50)	
VAS-PAIN (0–100 mm)	30 (10,40)	–
TJC	3 (1, 8)	–
SJC	2 (0, 4)	–
DAPSA	13.0 (5.9, 21.5)	–
cDAPSA	11.9 (4.8,19.8)	
Minimal disease activity, n (%)	14 (18.9%)	–
Uveitis, n (%)	5 (6.7%)	0(0%)
Psoriatic nail, n (%)	38 (51.3%)	21 (36.2%)
Dactylitis, n (%)	34 (45.9%)	–
Enthesitis, n (%)	4 (5.4%)	
Axial involvement, n (%)	12 (16.2%)	–
Treatment		
MTX, n (%)	63 (85.1%)	44 (75.8%)
NSAIDs, n (%)	12 (16.2%)	–
TNFi, n (%)	8 (10.8%)	3 (5.1%)
JAKi, n (%)	4 (5.4%)	3 (5.1%)
IL-17i, n (%)	1 (1.3%)	3 (5.1%)
MPO–DNA	0.351 (0.187, 0.652) ^*†^	0.200 (0.153, 0.429)^†^

Values are presented as n (%) for binary variables and mean ± SD or median (IQR) for continuous variables. *Significant difference when compared with patients with PsO, p < 0.05. ^†^Statistical significance when compared with HCs, p < 0.001. PsA duration: the duration of arthritis at baseline. Psoriasis, n (%): the number and percentage of patients with psoriatic skin lesion at baseline. MTX, methotrexate; NSAIDs, non-steroidal anti-inflammatory drugs; TNFi, tumor necrosis factor inhibitor; JAKi, Janus-activated kinase inhibitor; IL-17i, interleukin-17 inhibitor.

Before enrollment, 28 (37.8%) patients with PsA were treatment-naïve. All the remianing 46 patients with PsA ever received or were receiving disease modified anti-rheuamtic drugs (DMARDs). In details, methotrexate was used in 45 patients, salphasalazine in four patients, leflunomide in two patients, tumor necrosis factor inhibitors in six patients, and tofacitinib in one patient. Five patients ever exposed to non-steroidal anti-inflammaroty drugs.

### Serum MPO–DNA Level and Its Correlation With Disease Activity in Psoriatic Patients at Baseline

We observed higher serum level of MPO–DNA complex in both patients with PsA and PsO than that in healthy controls [median (IQR) 0.351 (0.187, 0.652) vs. 0.057 (0.049, 0.062); 0.200 (0.153, 0.429) vs. 0.057 (0.049, 0.062), *p* < 0.001 for both). The level of serum MPO–DNA complex in patients with PsA was even higher than that in patients with PsO [median (IQR) 0.351 (0.187, 0.652) vs. 0.200 (0.153, 0.429), *p* = 0.005] (**Table 1**). Binary logistic regression showed serum MPO–DNA was a risk factor for developing PsA (OR 5.541, *p* = 0.035). (**Supplementary Table S1**)

At baseline, there were 12 (16.2%), 28 (37.8%), 22 (29.7%), and 12 (16.2%) patients with PsA in remission, LDA, MDA, and HDA, respectively. Serum MPO–DNA complex was positively and linearly correlated with several indexes of disease activity, including TJC, SJC, PGA, EGA, VAS-pain, CRP, DAPSA, and cDAPSA (**Table 2**). In 34 patients with PsA with MDA/HDA, the DAPSA score was nicely correlated with serum MPO–DNA level (r = 0.438, *p* = 0.041; and r = 0.585, p = 0.046; respectively) however not in 12 patients with PsA with remission/LDA (**Table 3**). A significant correlation was also found between PASI score and serum MPO–DNA in 10 patients with PsO (r = 0.967, *p* < 0.001) however not in patients with PsA. Binary logistic regression also revealed serum MPO–DNA was a risk factor of MDA or HDA in patients with PsA (OR 187.2, p < 0.001). (**Supplementary Table S1**)

**Table 2:** Correlations of serum MPO–DNA level with disease activity index at baseline.

Item	PsA (n = 74)		PsO (n = 58)	
	r	p	r	p
ESR	0.188	0.111	0.152	0.255
CRP	0.25	0.033	0.288	0.028
PASI	0.147	0.211	0.479	<0.001
TJC	0.284	0.014	–	–
SJC	0.354	0.002	–	–
PGA (0–100 mm)	0.336	0.003	–	–
VAS-pain (0–100 mm)	0.365	0.001	–	–
DAPSA	0.409	<0.001	–	–
cDAPSA	0.404	<0.001		

Linear correlations of serum MPO–DNA complex with disease activity parameters in patients with PsO and PsA at baseline are presented by Pearson correlation coefficient (r).

**Table 3:** Correlation of serum MPO–DNA levels with DAPSA score in patients with PsA with different disease activity.

Disease Status	N	MPO–DNA, Median (IQR)	*p*	r
Remission	12	0.210 (0.183, 0.242)	0.039	−0.601
Low disease activity	28	0.236 (0.175, 0.429)	0.932	−0.017
Moderate disease activity	22	0.626 (0.432, 0.856)	0.041	0.438
High disease activity	12	0.568 (0.226, 0.769)	0.046	0.585
Total	74	0.351 (0.187, 0.652)	<0.001	0.409

### Changes of Disease Activity and Serum MPO–DNA in Patients With PsA After Treatment

Complete clinical data and serum at baseline, week 12, and week 24 of follow-up were available in 29 patients with PsA. Eight (27.5%) of them were treatment-naïve. Methotrexate was initiated in all patients at baseline. Combination therapy with tumor necrosis factor inhibitors, tofacitinib, cyclosporine, sulfasalazine, or non-steroidal anti-inflammatory drugs was applied in 11 patients. All the patients continued their medications unchanged throughout to week 24 except two patients (one initiated adalimumab, and one switched from adalimumab to secukinumab at week 12). At baseline, four (13.7%) patients with PsA were in minimal disease activity. According to DAPSA score, six (20.6%) patients were defined as with remission, 15 (51%) with LDA, seven (29.1%) with MDA, and one (3.4%) with HDA at week 12. After 12 weeks of treatment, PGA [median (IQR) 40 (20, 50) vs. 20 (10, 40), *p* = 0.047], VAS-pain score [median (IQR) 30 (10, 40) vs. 20 (10, 30), *p* = 0.034), and PSAI [median (IQR) 2.8 (0.3, 6.5) vs. 0.7 (0, 1.5) *p* = 0.013] of 29 patients were significantly improved. DAPSA score was decreased however statistically insignificant [15 (8.3, 23.4) vs. 8.4 (6.0, 14.4), *p* = 0.066] (**Table 3**). Meanwhile, MPO–DNA level was dramatically declined [0.416 (0.231, 0.846) vs. 0.231 (0.153, 0.474), *p* = 0.022]. More dramatic reduction of serum MPO–DNA in eight patients with PsA who reached both ACR50 and PASI50 than those who reached neither ACR50 nor PASI50 (n = 10) [0.532 (0.353, 0.741) vs. 0.064 (0.050, 0.206), *p* = 0.013] (**Supplementary Table S2**). During 24 weeks of follow-up, all disease activity measures were continually decreasing (**Table 4**). Five (17.2%) patients with PsA achieved both ACR70 and PASI75 responses at week 24.

**Table 4:** Treatment response and changes of serum MPO–DNA of 29 patients with PsA.

Items	Baseline	Week 12	Week 24
TJC	3.0 (1.0, 8.0)	3.0 (1.0, 5.0)	2.0 (0.2, 3.7)
SJC	2.0 (1.0, 6.0)	2.0 (1.0, 3.0)	1.0 (0, 3.0)
ESR, mm/h	13.0 (7.7, 22.7)	8 (4.0, 14.0)	10 (2.0, 16.0)
CRP, mg/L	3.0 (1.6, 11.8)^†^	2.6 (1.3, 5.0)	2.3 (1.0, 4.8)
PGA (0–100 mm)	40 (20, 50)^*††^	20 (10, 40)^††^	10 (10, 27)
VAS-pain (0–100 mm)	30 (10, 40)^*††^	20 (10, 30)^††^	10 (10, 20)
PASI	2.8 (0.3, 6.5)^*†^	0.7(0, 1.5)	0.3 (0, 1.5)
DAPSA	15 (8.3, 23.4)^†^	8.4 (6.0, 14.4)	6.1 (3.4, 13.7)
Serum MPO–DNA level	0.416 (0.231, 0.846)^*^	0.231 (0.153, 0.474)	–
Minimal disease activity, n (%)	4 (13.7%)	6 (20.6%)	12 (41.3%)
ACR50, n (%)	–	13 (44.8%)	15 (51.7%)
PASI50, n (%)	–	14 (48.2%)	17 (58.6%)
ACR70, n (%)	–	4 (13.7%)	7 (24.1%)
PASI75, n (%)	–	11 (37.9%)	14 (48.2%)
ACR50/PASI50, n (%)	–	8 (27.5%)	13 (44.8%)
ACR70/PASI75, n (%)	–	3 (10.3%)	5 (17.2%)

Values are presented as n (%) for binary variables or median (IQR) for continuous variables. TJC, tenderness joint count; SJC, swollen joint count; ESR, erythrocyte sediment rate; CRP, C-reactive protein; PGA, patient global assessment; EGA, examiner global assessment; DAPSA, disease activity in psoriatic arthritis score; ACR50/70, 50/70% improvement in terms of American College of Rheumatology response criteria; PASI50/75, 50/75% reduction in PASI score.

^*^Statistical significance at the level of 0.05 when compared with patients at week 12.

^†^Statistical significance at the level of 0.05 when compared with patients at week 24.

^††^Statistical significance at the level of 0.001 when compared with patients at week 24.

### Predictive Values of Serum MPO–DNA for Therapeutics Response

**Figure 1:**
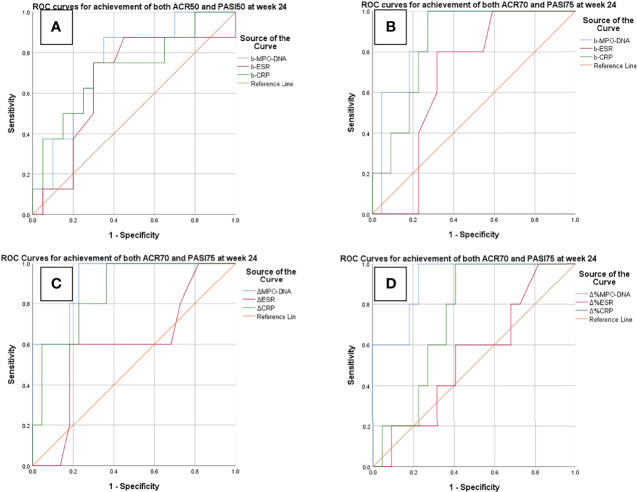
ROC curves for predictions of treatment response at weeks 12 and 24. **(A)** Baseline serum MPO–DNA, ESR, and CRP to predict achievement of both ACR50 and PASI 50 at week 12. **(B–D)** Baseline and changes of MPO–DNA, ESR, and CRP to predict achievement of ACR70 and PASI75. b-MPO–DNA/ESR/CRP, serum MPO–DNA complex/ESR/CRP at baseline; ΔMPO–DNA/ESR/CRP, reduction of serum MPO–DNA/ESR/CRP at week 12 in absolute value; ΔMPO–DNA/ESR/CRP%, reduction of serum MPO–DNA/ESR/CRP at week 12 from baseline in percentage.

The ROC analysis showed that the baseline serum MPO–DNA predicted the achievement of both ACR50 and PASI50 response at week 12 as well as achievement of both ACR70 and PASI75 response at week 24. The AUC of baseline MPO–DNA for therapeutic response at week 12 (AUC = 0.74; 95% CIs, 0.55–0.93) and week 24 (AUC = 0.88; 95% CIs, 0.74–1) was greater than that of ESR and CRP (**Table 5** and **Figure 1**). The optimal cutoffs of baseline serum MPO–DNA to predict treatment outcomes at weeks 12 and 24, determined by the Youden index, were 0.416 (positive predictive value, PPV = 0.5; negative predictive value, NPV = 0.92) and 0.824 (PPV = 0.5, NPV = 0.95), respectively (**Table 6**). The ROC analysis also revealed that decrease of serum MPO–DNA levels at week 12 predicted the achievement of both ACR70 and PASI75 at week 24. Regarding the reduction of serum MPO–DNA at 12 weeks from baseline (ΔMPO–DNA), the AUC of ΔMPO–DNA in absolute value and in percentage (AUC = 0.92; 95% CIs, 0.8–1; AUC = 0.92; 95% CIs, 0.8–1) were significantly greater than the changes of ESR and CRP (**Table 5** and **Figure 1**). There were significant differences between AUCs for ΔMPO–DNA and ΔESR in absolute value (AUC difference = 0.31; *p* = 0.036) and AUCs for ΔMPO–DNA and ΔESR in percentage (AUC difference = 0.373; *p* = 0.02). The optimal cutoff values for ΔMPO–DNA and ΔMPO–DNA%, determined by the Youden index, were 0.34 (PPV = 0.44, NPV = 0.95) and 56.3% (PPV = 0.4, NPV = 0.94), respectively (**Table 6**). Both the baseline and changes of serum MPO–DNA failed to predict the achievement of minimal disease activity at 12 or 24 weeks by ROC analysis in the study (**Supplementary Tables S3**, **S4** and **Figure S1**).

**Table 5:** AUCs for treatment outcomes at weeks 12 and 24.

Measures	AUC for both ACR50 and PASI50 at week 12				AUC for both ACR70 and PASI75 at week 24			
	AUC (95%CIs)	*p*	AUC difference	*p*	AUC (95%CIs)	*p*	AUC difference	*p*
b-MPO–DNA	0.74 (0.55–0.93)	0.047	Reference	–	0.88 (0.74–1)	0.009	Reference	
b-ESR	0.66 (0.42–0.89)	0.195	0.08	0.60	0.68 (0.47–0.81)	0.224	0.20	0.14
b-CRP	0.72 (0.5–0.94)	0.075	0.02	0.877	0.84 (0.69–0.99)	0.018	0.03	0.76
ΔMPO–DNA	–	–	–	–	0.92 (0.8–1)	0.004	Reference	
ΔESR	–	–	–	–	0.6 (0.31–0.88)	0.143	0.31	0.036
ΔCRP	–	–	–	–	0.86 (0.7–1)	0.013	0.05	0.42
ΔMPO–DNA%	–	–	–	–	0.92 (0.8–1)	0.004	Reference	
ΔESR%	–	–	–	–	0.54 (0.28–0.8)	0.134	0.373	0.02
ΔCRR%	–	–	–	–	0.73 (0.54–0.92)	0.097	0.182	0.184

Values are presented as areas under curve (95% CIs). Treatment outcomes are referred to the achievement of both ACR50 and PASI50 at week 12 and the achievement of both ACR70 and PASI75 at week 24. Abbreviations: b-MPO–DNA/ESR/CRP, serum MPO–DNA complex/ESR/CRP at baseline; ΔMPO–DNA/ESR/CRP, reduction of serum MPO–DNA/ESR/CRP at 12 weeks in absolute value; ΔMPO–DNA/ESR/CRP%, reduction of serum MPO–DNA/ESR/CRP at 12 weeks in percentage.

**Table 6:** Cutoff points of serum biomarkers and relevant sensitivity, specificity, PPV, and NPV when predicting ACR and PASI improvement at weeks 12 and 24.

Item	Youden index	Cutoff	Sen	Spe	PPV	NPV
Week 12						
b-MPO–DNA	0.525	0.416	0.875	0.65	0.5	0.92
b-ESR	0.45	16.5	0.75	0.7	0.5	0.875
b-CRP	0.45	4.44	0.75	0.7	0.5	0.875
Week 24						
b-MPO–DNA	0.618	0.824	0.8	0.818	0.5	0.95
ΔMPO–DNA	0.618	0.34	0.8	0.818	0.44	0.95
ΔMPO–DNA%	0.618	56.3%	0.8	0.818	0.4	0.94
b-ESR	0.482	18.5	0.8	0.318	0.36	0.94
ΔESR	0.418	6.5	0.6	0.818	0.42	0.9
ΔESR%	0.191	30.1%	0.6	0.591	0.25	0.87
b-CRP	0.573	9.3	0.8	0.773	0.57	0.94
ΔCRP	0.573	3.7	0.8	0.773	0.44	0.94
ΔCRP%	0.436	47.3%	0.8	0.636	0.31	0.93

Sen, sensitivity; Spe, specificity; PPV, positive predictive value; NPV, negative predictive value; b-MPO–DNA/ESR/CRP, serum MPO–DNA complex/ESR/CRP at baseline; ΔMPO–DNA/ESR/CRP, reduction of serum MPO–DNA/ESR/CRP at 12 week in absolute value; ΔMPO–DNA/ESR/CRP%, reduction of serum MPO–DNA/ESR/CRP at 12 weeks in percentage.

## Discussion

Emerging evidence has indicated that NETosis is involved in the pathogenesis of autoimmune diseases, including PsO. NETosis could contribute to the inflammation by activating the epidermal TLR4/IL-36R cross-talk and stimulating inflammatory mediators, such as interferon-α and interferon-β, in PsO (20). However, few studies ever focused on the role of NETosis in PsA so far. Previous research studies proposed that LL37 produced by NETosis as the antigen triggering the inflammation in PsA may be a different pathway of NETosis involvement from rheumatoid arthritis or systemic lupus erythematosus (21). In this study, we demonstrated that the NETosis marker, MPO–DNA complex in serum, was markedly elevated in patients with PsA and PsO and positively correlated with psoriatic disease burden. Moreover, serum MPO–DNA may be used as a biomarker to predict the therapeutic response in patients with PsA.

The level of NETosis can be evaluated directly by observation or indirectly by measuring its products. The MPO–DNA complex is one of the products of neutrophils with high objectivity and specificity to NETosis (22). Therefore, we measured serum MPO–DNA complex to reflect the extent of spontaneous NETosis in patients with PsO/PsA. Increased MPO–DNA complex in serum has been found in many autoimmune diseases such as rheumatoid arthritis, Behcet’s disease, and ANCA-associated vasculitis (12, 13, 23). In the present study, we observed the same phenomena in patients with psoriasis. In particular, we observed higher serum MPO–DNA levels in PsA than that in patients with PsO, indicating more spontaneous NETosis and probably heavier inflammatory burdens in patients with PsA.

MPO–DNA complex had its advantages for serving as an inflammatory biomarker. First, as the products of NETosis, detecting the serum MPO–DNA complex could offer extra information about the inflammatory burden of psoriatic disease. Second, our study revealed a positive correlation of serum MPO–DNA level with disease activity parameters of psoriatic diseases, which was similar to research studies of Wojcik et al. and Leon et al. (24, 25) In the current analysis, we did not find the correlation between PASI score and serum MPO–DNA in patients with PsA. We reckon that NETosis takes part in different pathogenesis pathways in psoriasis and PsA. However, the inconsistent findings available so far calls for more studies to confirm the correlation between NETosis remnants and the disease activity of psoriatic diseases.

We observed that the more active the disease, the better the correlation of serum MPO–DNA levels with DAPSA score in PsA. It is interesting that we observed a negative correlation between serum MPO–DNA levels and DAPSA score in patients with PsA with remission. Because the serum MPO–DNA complex levels in remissive patients with PsA were significantly lower than those in active patients with PsA, the correlation of serum MPO–DNA and DAPSA score in patients with PsA in remission may be less meaningful. 

In addition to the correlations with disease activity, we observed decrease of serum MPO–DNA level when patients with PsA were improved after treatment. The patients with PsA who responded well to treatment showed a more considerable reduction of serum MPO–DNA complex level. The change of serum MPO–DNA levels may reflect the changes of both skin lesion and arthritis, whereas the change of traditional parameters can only mirror the change of one aspect of PsA. As NETosis is a vital part of the pathogenesis in psoriatic disease, the evaluation of serum NETosis products could directly reflect the disease burden. Similar phenomena were observed in other inflammatory diseases, such as RA and adult-onset Still’s disease (26, 27).

In the present study, the medications of 29 patients with PsA were barely changed during 24 weeks of follow-up. The current ROC analysis indicated that the serum MPO–DNA level could predict the treatment response of patients with PsA. The relatively higher MPO–DNA level at baseline or dramatically decreased MPO–DNA indicates greater probability for a patients with PsA to achieve improvements at 12 and 24 weeks. The predictive effect of baseline serum MPO–DNA was better than ESR and CRP. This means that monitoring serum MPO–DNA may help to adjust the treatment regimen and avoid inappropriate treatment attempts. Serum MPO–DNA can be used as an inflammatory biomarker to guide the treatment in patients with PsA.

We are aware of some limitations of the study. First, the size of sample was not specially assessed. All the patients with available data were included. Second, because of the limited sample size, we are not able to conduct subgroup analysis stratified by different medications. The effects on NETosis exerted by different DMARDs in psoriatic disease remain unclear. Further research is needed to specific impacts on NETosis caused by different DMARDs. Third, we did not replicate the findings in an independent cohort. We will continue the validation work in the future study.

## Conclusions

The study revealed increased serum MPO–DNA complex in patients with psoriasis and its correlation with PsA disease activity. NETosis is involved in the psoriatic inflammation. Serum MPO–DNA complex possesses the potential as a useful biomarker to predict the therapeutic response in patients with PsA.

## Data Availability Statement

The raw data supporting the conclusions of this article will be made available by the authors, without undue reservation.

## Ethics Statement

The studies involving human participants were reviewed and approved by Ethics Committee of the Peking University First Hostpital. The patients/participants provided their written informed consent to participate in this study. Written informed consent was obtained from the individual(s) for the publication of any potentially identifiable images or data included in this article.

## Author Contributions

GL and ZZ conceived and designed the study. BL, YW, and ZS collected the data. BL and XY performed the ELISA analysis. BL performed the statistical analysis and drafted the manuscript. ZZ also critically revised the manuscript. All authors contributed to the article and approved the submitted version.

## Conflict of Interest

The authors declare that the research was conducted in the absence of any commercial or financial relationships that could be construed as a potential conflict of interest.

## Publisher’s Note

All claims expressed in this article are solely those of the authors and do not necessarily represent those of their affiliated organizations, or those of the publisher, the editors and the reviewers. Any product that may be evaluated in this article, or claim that may be made by its manufacturer, is not guaranteed or endorsed by the publisher.

## Supplementary Material

Supplementary Table 1 Binary logistic regressions for PsO/PsA and moderate to high disease activity of PsA.

Supplementary Table 2 Comparison of baseline clinical characteristics and ΔMPO–DNA of different groups of patients with PsA divided by different treatment response at 12 weeks. Group1: Patients with PsA who achieved both ACR50 and PASI50 response at week 12, n = 8 (27.5%); Group2: Patients with PsA who achieved either ACR50 or PASI50 response at week 12, n = 11 (37.9%); Group3: Patients with PsA who achieved neither ACR50 nor PASI50 response at week 12, n = 10 (34.4%). ^*^Statistical significance at the level of 0.05 when compared with group2. ^†^Statistical significance at the level of 0.05 when compared with group3.

Supplementary Table 3 AUCs for achievement of minimal disease activity at 12 and 24 weeks. Values are presented as areas under curve (95% CIs). b-MPO–DNA/ESR/CRP, serum MPO–DNA complex/ESR/CRP at baseline; ΔMPO–DNA/ESR/CRP, reduction of serum MPO–DNA/ESR/CRP at week 12 in absolute value; ΔMPO–DNA/ESR/CRP%, reduction of serum MPO–DNA/ESR/CRP at week 12 from baseline in percentage.

Supplementary Table 4 Cutoff points of serum biomarkers and relevant sensitivity, specificity, PPV, and NPV. Sen, sensitivity; Spe, specificity; PPV, positive predictive value; NPV, negative predictive value; b-MPO–DNA/ESR/CRP, serum MPO–DNA complex/ESR/CRP at baseline; ΔMPO–DNA/ESR/CRP, reduction of serum MPO–DNA/ESR/CRP at 12 weeks in absolute value; ΔMPO–DNA/ESR/CRP%, reduction of serum MPO–DNA/ESR/CRP at 12 weeks in percentage.

## Abbreviations

PsA, psoriatic arthritis; PsO, psoriasis; NETs, neutrophil extracellular traps; ROC, receiver operating characteristic; AUC, area under the curve; CASPAR, classification criteria for psoriatic arthritis; DAPSA, disease activity in psoriatic arthritis; PASI, psoriasis area and severity index; LDA, low disease activity; MDA, moderate disease activity; HDA, high disease activity; TJC, tenderness joint count; SJC, swollen joint count; PGA, patients global assessment; ESR, erythrocyte sedimentary rate; CRP, C-reactive protein; HRP, horseradish peroxidase; OD, optical density.
