# Supplementary material for: NETosis in Psoriatic Arthritis: Serum MPO–DNA Complex Level Correlates With Its Disease Activity
Source: Front Immunol. 2022 Jun 14;13:911347. doi: 10.3389/fimmu.2022.911347 (PMC9238436; doi:10.3389/fimmu.2022.911347)
Supplement: Supplementary Table 1 — Binary logistic regressions for PsO/PsA and moderate to high disease activity of PsA. [file DataSheet_1.docx]

Supplementary table S1 Binary logistic regressions for PsO/PsA and moderate to high disease activity of PsA

| Variables | PsO/PsA |  |  | MDA/HDA |  |  |
| --- | --- | --- | --- | --- | --- | --- |
|  | *p* | OR | 95% CIs | *p* | OR | 95% CIs |
| Age | 0.278 | 1.018 | 0.986-1.051 | 0.475 | 0.983 | 0.937-1.031 |
| Gender | 0.232 | 1.626 | 0.773-3.068 | 0.104 | 3.432 | 0.776-15.171 |
| Psoriatic nail | 0.051 | 2.184 | 0.998-4.781 | 0.003 | 10.292 | 2.177-48.666 |
| b-MPO-DNA | 0.035 | 5.541 | 1.129-27.183 | ＜0.001 | 187.298 | 12.528-2800.103 |
| b-ESR | 0.900 | 1.003 | 0.952-1.057 | 0.107 | 1.053 | 0.989-1.121 |
| b-CRP | 0.065 | 1.079 | 0.995-1.170 | 0.915 | 0.997 | 0.942-1.055 |

Supplementary table S2 Comparison of baseline clinical characteristics and ΔMPO-DNA of different groups of PsA patients divided by different treatment response at 12-week

| Item | Group1  (n=8) | Group2  (n=11) | Group3  (n=10) | *P* |
| --- | --- | --- | --- | --- |
| Male, n (%) | 4 (50%) | 7 (64%) | 5 (50%) | 0.774 |
| Age, mean (SD), yrs | 51 (11) | 49 (9) | 54 (15) | 0.633 |
| Disease duration, yrs | 4.3 (1.6, 5.2) | 4.7 (1.2, 7.5) | 4.6 (2.6, 9.4) | 0.834 |
| Treatment-naïve,n (%) | 3 (38%) | 2 (18%) | 3 (30%) | 0.635 |
| TJC | 9.0 (5.0, 11.0)* | 1.0 (0.5, 2.5) | 6.5 (1.3, 8.0) | 0.014 |
| SJC | 4.5 (3.0, 7.2) | 1.0 (0, 3.0) | 2.0 (1.3, 5.3) | 0.075 |
| ESR, mm/h | 22 (26.0, 17.2) | 10 (8, 16.5) | 11 (4.8, 20.3) | 0.349 |
| CRP, mg/L | 9.1 (1.9, 25.8) | 3.6 (6.5) | 3.3 (7.4) | 0.495 |
| PGA (0-100mm) | 40 (36, 52) | 20 (15, 50) | 30 (20, 50) | 0.401 |
| EGA (0-100mm) | 35 (30,40) | 20 (10, 40) | 23 (20, 45) | 0.411 |
| VAS-pain (0-100mm) | 40 (30, 42)* | 10 (10, 25) | 35 (20, 68) | 0.055 |
| PASI | 3.8 (2.7, 7.0)^†^ | 3.8 (1.0, 6.9) ^†^ | 0.3 (0, 2.1) | 0.032 |
| DAPSA | 22.6 (17.2, 27.4)^*^ | 8.3 (5.0, 10.7) | 16.5(8.7, 23.6) | 0.021 |
| b-MPO-DNA | 0.859 (0.610, 1.027)^*†^ | 0.309(0.230, 0.674) | 0.279(0.191, 0.398) | 0.015 |
| ΔMPO-DNA | 0.532 (0.353, 0.741) ^*†^ | 0.027(0.073, 0.206) | 0.064(0.050, 0.113) | 0.025 |

Group1: PsA patients who achieved both ACR50 and PASI50 response at week 12, n=8 (27.5%); Group2: PsA patients who achieved either ACR50 or PASI50 response at week 12, n=11 (37.9%); Group3: PsA patients who achieved neither ACR50 nor PASI50 response at week 12, n=10 (34.4%)

^*^Statistical significance at the level of 0.05 when compared with group2

^†^Statistical significance at the level of 0.05 when compared with group3.

Supplementary table S3 AUCs for achievement of minimal disease activity at 12 and 24 weeks.

| Measures | AUC for minimal disease activity at 12-week | | | | AUC for minimal disease activity at 24-week | | | |
| --- | --- | --- | --- | --- | --- | --- | --- | --- |
|  | AUC (95%CIs) | *p* | AUC difference | *p* | AUC (95%CIs) | *p* | AUC difference | *p* |
| b-MPO-DNA | 0.74(0.55-0.92) | 0.073 | reference |  | 0.54(0.32-0.76) | 0.696 | Reference |  |
| b-ESR | 0.75(0.51-1) | 0.057 | 0.01 | 0.91 | 0.53(0.31-0.76) | 0.733 | 0.005 | 0.96 |
| b-CRP | 0.62(0.32-0.92) | 0.356 | 0.11 | 0.46 | 0.486(0.25-0.71) | 0.903 | 0.03 | 0.86 |
| ΔMPO-DNA |  |  |  |  | 0.46(0.23-0.68) | 0.733 | Reference |  |
| ΔESR |  |  |  |  | 0.52(0.29-0.74) | 0.845 | 0.01 | 0.92 |
| ΔCRP |  |  |  |  | 0.44(0.22-0.67) | 0.643 | 0.01 | 0.92 |
| Δ%MPO-DNA |  |  |  |  | 0.42(0.2-0.65) | 0.526 | reference |  |
| Δ%ESR |  |  |  |  | 0.36(0.15-0.58) | 0.252 | 0.05 | 0.68 |
| Δ%CRR |  |  |  |  | 0.45(0.23-0.68) | 0.714 | 0.02 | 0.83 |

Values are presented as areas under curve (95% CIs). Abbreviations: b-MPO-DNA/ESR/CRP, Serum MPO-DNA complex/ESR/CRP at baseline; ΔMPO-DNA/ESR/CRP, reduction of serum MPO-DNA/ESR/CRP at week 12 in absolute value; ΔMPO-DNA/ESR/CRP%, reduction of serum MPO-DNA/ESR/CRP at week 12 from baseline in percentage.

Supplementary table S4 Cut-off points of serum biomarkers and relevant sensitivity, specificity, PPV and NPV.

| Item | Youden index | Cut-off | Sen | Spe | PPV | NPV |
| --- | --- | --- | --- | --- | --- | --- |
| For week 12 (mda) | | | | | | |
| b-MPO-DNA | 0.47 | 0.38 | 0.636 | 0.383 | 0.38 | 0.93 |
| b-ESR | 0.53 | 7.5 | 0.86 | 0.77 | 0.57 | 0.9 |
| b-CRP | 0.26 | 3.2 | 0.59 | 0.67 | 0.25 | 0.81 |
| For week 24 (mda) | | | | | | |
| b-MPO-DNA | 0.18 | 0.38 | 0.6 | 0.583 | 0.53 | 0.68 |
| ΔMPO-DNA | 0.18 | 0.07 | 0.58 | 0.6 | 0.41 | 0.58 |
| ΔMPO-DNA% | 0.18 | 32.9% | 0.58 | 0.6 | 0.46 | 0.64 |
| b-ESR | 0.06 | 8.5 | 0.73 | 0.33 | 0.44 | 0.57 |
| ΔESR | 0.15 | 1.5 | 0.73 | 0.42 | 0.38 | 0.5 |
| ΔESR% | 0.26 | 27.5% | 0.67 | 0.6 | 0.57 | 0.71 |
| b-CRP | 0.1 | 3.8 | 0.5 | 0.6 | 0.46 | 0.6 |
| ΔCRP | 0.1 | 2.4 | 0.5 | 0.6 | 0.46 | 0.6 |
| ΔCRP% | 0.15 | 58.6% | 0.42 | 0.73 | 0.57 | 0.9 |

Abbreviations: Sen, sensitivity; Spe, specificity; PPV, positive predictive value; NPV, negative predictive value; b-MPO-DNA/ESR/CRP, Serum MPO-DNA complex/ESR/CRP at baseline; ΔMPO-DNA/ESR/CRP, reduction of serum MPO-DNA/ESR/CRP at 12-week in absolute value; ΔMPO-DNA/ESR/CRP%, reduction of serum MPO-DNA/ESR/CRP at 12-week in percentage.

**
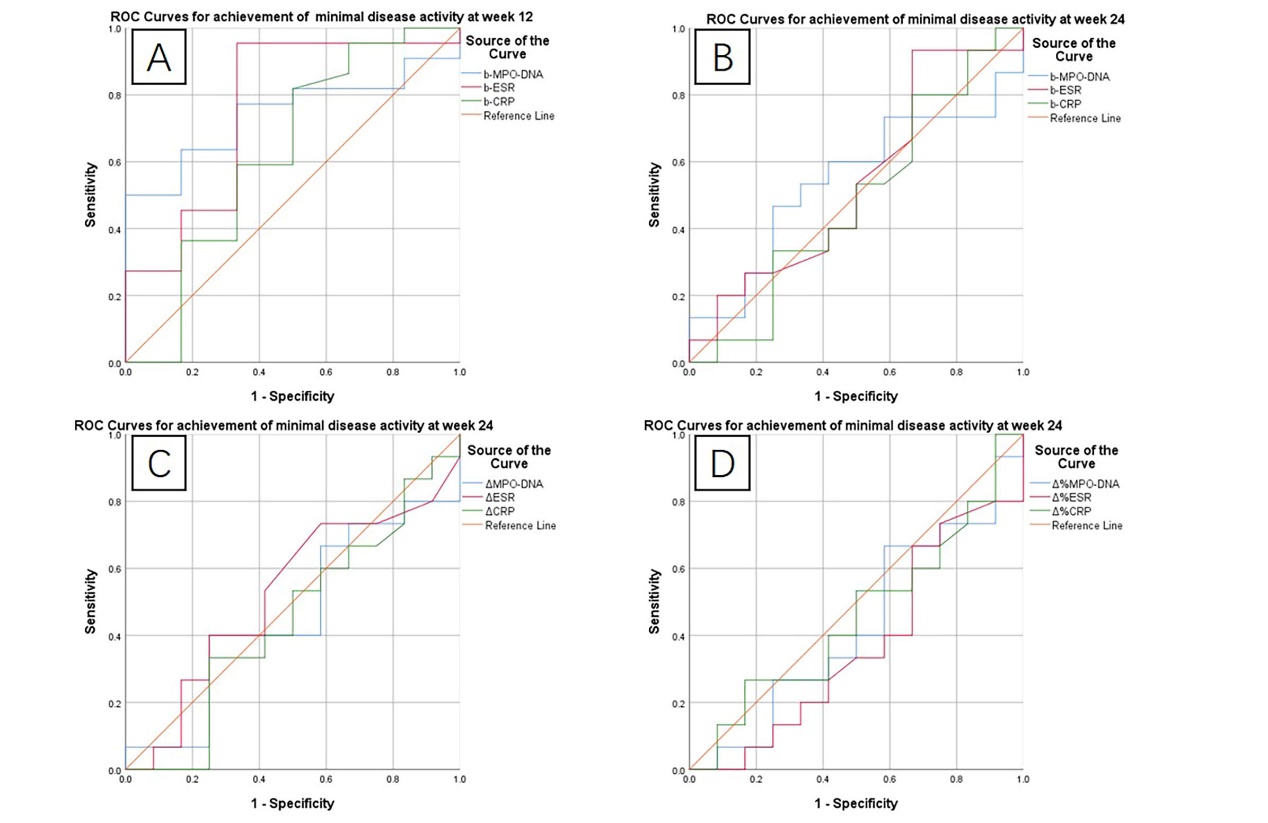
**

**Supplementary Fig. S1**. ROC curves for achievement of minimal disease activity at week 12 and 24. **A**. Week 12; **B-D**. Week 24. Abbreviations: b-MPO-DNA/ESR/CRP, Serum MPO-DNA complex/ESR/CRP at baseline; ΔMPO-DNA/ESR/CRP, reduction of serum MPO-DNA/ESR/CRP at week 12 in absolute value; ΔMPO-DNA/ESR/CRP%, reduction of serum MPO-DNA/ESR/CRP at week 12 from baseline in percentage.
